# Effect of Intravenous Dexamethasone Dose on the Occurrence of Rebound Pain after Axillary Plexus Block in Ambulatory Surgery

**DOI:** 10.3390/jcm12134310

**Published:** 2023-06-27

**Authors:** Nassim Touil, Athanassia Pavlopoulou, Simon Delande, Pierre Geradon, Olivier Barbier, Xavier Libouton, Patricia Lavand’homme

**Affiliations:** 1 Department of Anaesthesiology, Cliniques Universitaires Saint-Luc, Catholic University of Louvain, B-1200 Brussels, Belgium; 2 Department of Orthopaedic Surgery, Cliniques Universitaires Saint-Luc, Catholic University of Louvain, B-1200 Brussels, Belgium

**Keywords:** loco-regional anesthesia, rebound pain, dexamethasone, ambulatory surgery

## Abstract

Rebound pain (RP) remains a challenge in ambulatory surgery, characterized by severe pain upon resolution of a peripheral nerve block (PNB). Intravenous (IV) administration of Dexamethasone (DEXA) potentiates PNB analgesic effect and reduces RP incidence although preventive effective dose remains undetermined. This retrospective analysis evaluates the preventive effect of IV DEXA on RP in outpatients undergoing upper limb surgery under axillary block. DEXA was divided into high (HD > 0.1 mg/kg) or low (LD < 0.1 mg/kg) doses. RP was defined as severe pain (NRS ≥ 7/10) within 24 h of PNB resolution. DEXA HD and LD patients were matched with control patients without DEXA (*n* = 55) from a previous randomized controlled study. Records of 118 DEXA patients were analyzed (DEXA dose ranged from 0.05 to 0.12 mg/kg). Intraoperative IV DEXA was associated with a significant reduction of the pain felt when PNB wore off as well as to a significant reduction of RP incidence (*n* = 27/118, 23% vs. 47% in controls, *p* = 0.002) with no effect related to the dose administered (*p* = 0.053). Our results support the administration of intraoperative DEXA as a preventive measure to reduce the occurrence of RP.

## 1. Introduction

Regional anesthesia offers several advantages in ambulatory setting, including high-quality perioperative analgesia and faster discharge from the hospital.

Peripheral nerve blocks (PNBs) using different techniques [1] are extensively used in orthopedic limb surgeries as they ease the surgical procedure and they improve patient satisfaction [2], particularly in terms of long-lasting postoperative analgesia without systemic drug side effects [1]. However, following PNB resolution, a rapid increase in pain intensity commonly referred to as “rebound pain” (RP) may occur. The phenomenon is observed within the first 24 to 48 h of the PNB dissipation. Occurring outside of health care setting, it is actually considered as a clinically relevant problem [3,4]. Further, RP is the most frequent factor of dissatisfaction reported by the patients following PNB. The RP phenomenon is frequent as it may affect nearly 40% to 50% of orthopedic patients operated under PNB [2,5,6].

For the aforementioned reasons, the prevention of RP occurrence has become a priority, particularly in ambulatory surgery setting. Several risk factors have been found to be associated with the phenomenon such as younger age, female gender, high catastrophizing mind set, bone surgery and the absence of perioperative administration of dexamethasone [5,6]. Dexamethasone (DEXA), a synthetic corticosteroid, is widely used in anesthesia practice for its well-known perioperative analgesic and antiemetic properties [7]. Moreover, DEXA also serves as an adjuvant to PNB to increase the block duration and the analgesic related effect [8]. PNB prolongation has been observed after the administration of DEXA by either the perineural or intravenous (IV) route [9]. The literature suggests that 4 mg of DEXA may be the optimal perineural dose to potentiate PNB, whereas the optimal intravenous dose is likely higher but still remains undetermined [10]. However, the intravenous administration should be preferred because perineural use may cause delayed neurotoxicity and the perineural route is still off-label [9]. Several studies have reported a reduction of RP incidence after PNB dissipation when perioperative DEXA was administered by either the perineural [11,12] or intravenous route [5,13]. To date, the dose-related preventive effect of intravenous DEXA on RP has not been evaluated.

The main objective of this retrospective study was to assess the occurrence of RP when perioperative IV DEXA was administered at analgesic (dose higher than 0.1 mg/kg [14] and/or antiemetic doses [7,15] (i.e., doses ranging between 4 and 10 mg) in ambulatory patients undergoing upper limb orthopedic surgery under axillary plexus block. The secondary objective was to evaluate a potential dose-related preventive effect of IV DEXA on the development of RP.

## 2. Materials and Methods

This single-center retrospective cohort study was conducted at the Cliniques Universitaires Saint-Luc (Brussels, Belgium). It is a retrospective analysis of data collected prospectively in the context of Quality Reporting in the Out-Patients Unit. The data were collected over a period of 1 year, between January 2021 and February 2022, from patients’ files and perioperative questionnaire-based sources.

### 2.1. Recruitment

**Figure 1:**
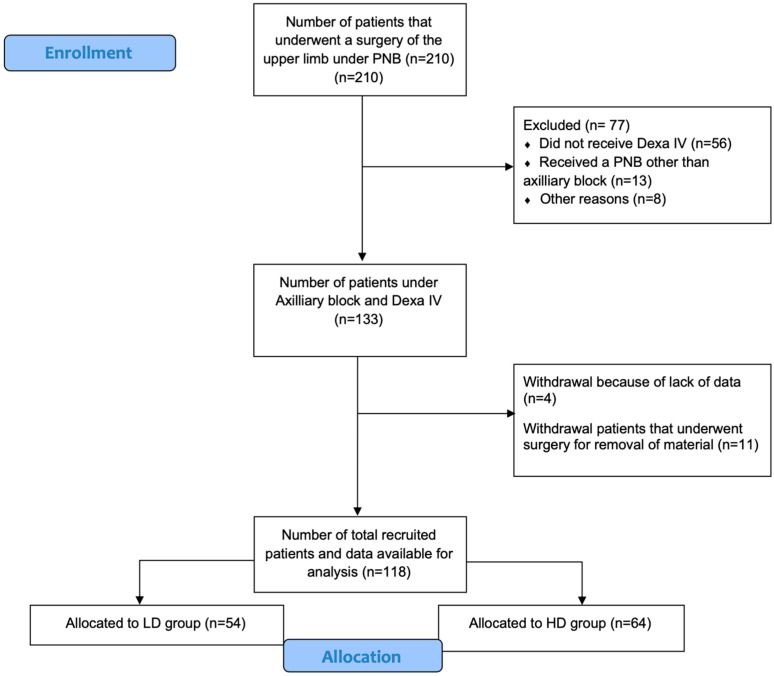
Flow Diagram. Abbreviations: PNB—Peripheral Nerve. Block; Dexa IV—Intravenous Dexamethasone; LD—Low dose; HD—High dose.

Ambulatory adult patients (18 to 80 years old) who underwent elective upper limb surgery (elbow and below) under axillary plexus block between January 2021 and February 2022 were included (Figure 1). Only patients who had complete perioperative data records were considered. Eligible patients had received an intravenous DEXA dose left to the discretion of the anesthesiologist in charge. Intraoperative IV DEXA was administered either as an antiemetic or as an analgesic adjuvant to the PNB, i.e., IV doses ranging from 4 to 10 mg. All the patients underwent axillary PNB under real-time ultrasound guidance by a trained anesthesiologist. The control group was composed of patients included in a previous prospective study on rebound pain, who had not received DEXA [6]. All the patients, in the control group and those included between January 2021 and February 2022, received the same type of PNB, i.e., an axillary plexus block. Each axillary plexus was performed using a 50:50 mixture of ropivacaine (0.5%) and mepivacaine (1%). The reuse of prospective data did not require the consent of the patients concerned for the retrospective analysis by the ethics committee. The authors are not sure that it should be included in the manuscript, but it should only figure in the answers to the reviewers’ concerns.

All the patients benefited from the same perioperative analgesic protocol using multimodal analgesia. Patients who had contra-indication to the analgesics used in the perioperative multimodal analgesic protocol (i.e., paracetamol or non-steroidal analgesic use) were not included in the analysis. Patients who received IV DEXA were matched with patients of the control group regarding age, sex and type of surgery (bone or soft tissues surgery) (Table 1).

### 2.2. Data Collection

To improve the management of postoperative pain by better focusing on patients at risk of developing severe acute postoperative pain when back home, a set of preoperative questionnaires is commonly proposed to the patients to all the ambulatory patients including those undergoing regional anesthesia for ambulatory orthopedic surgery. All the questionnaires are completed on a voluntary basis.

All the patients included in the study had complete perioperative records. Preoperative data collected on day 0 included preoperative pain at rest, pain on movement and pain overnight at the surgical site, using a numerical rating scale (NRS) from 0 to 10 (where 0 = no pain and 10 = worst pain). Preoperative medications, including pain relievers, were also noted. Patients also completed a pre-operative questionnaire that had been developed in 2019 as part of a previous prospective study [6]. The anesthesia team continued to use this questionnaire in their practice to help prevent severe acute postoperative pain at home, mainly related to a rebound pain phenomenon.

Patients were therefore asked to answer a series of preoperative questions such as the French version of the Pain Catastrophizing Scale (PCS) questionnaire, which assess negative thoughts related to pain (i.e., rumination, amplification and helplessness) on a scale from 0 to 52 [14]. Patients were classified as high catastrophizers if they scored higher than the 75th percentile.

The French version of the Central Sensitization Inventory (CSI) was used to measure the main somatic and emotional complaints associated with central sensitization. A validated short-form was used as CSI-9 (i.e., 9 questions derived from the original 40 questions, with a cut-off value of 20 on a 0–36 scale) to distinguish patients presenting with a preoperative positive central sensitization [16]. Finally, the presence of a neuropathic component in the preoperative pain at the surgical site was assessed using the “Douleur Neuropathique 4” (DN4) questionnaire (cutoff value of 4 on a 10-point score) [17].

Before the surgical incision, the effectiveness of the sensory blockade of the axillary plexus was evaluated by a cold test (ether test) in the different territories. Only patients who presented with fully effective axillary PNB were considered for inclusion in the study (Figure 1). For various reasons (surgeon’s request, patient comfort, PNB failure …), 8 patients who received general anesthesia during the procedure in addition to the axillary plexus were excluded. All the patients were operated upon by two orthopedic surgeons (O.B. and X.L). A standardized optimal perioperative multimodal analgesic treatment was applied to all the patients, including intraoperative administration of paracetamol (1 g) and intraoperative ketorolac (0.5 mg/kg). In the recovery room, when the postoperative pain score (NRS) was >3/10, intraveinous tramadol (2 mg/kg) was administered. Patients were discharged with written recommendations for the use of standard postoperative oral analgesics, i.e., ibuprofen 400 mg/6 h, paracetamol 3 g/24 h and, if necessary, tramadol as a rescue analgesic (1–2 mg/kg; Maximum 400 mg/day). The patients were also asked to note in a pain diary the time when the axillary block wears off, as well as the intensity of the pain felt (NRS, 0–10) at that time and the analgesics intake. All the patients were contacted by phone call on day 1 at 24 h after surgery (regular telephone call for quality audit purpose) by a hospital nurse. Postoperative pain intensity was questioned, including average and maximal pain on a NRS scale from 0 to 10.

The definition of RP was the same we used in our previous study [6], i.e., the same used in the control group. RP was defined as severe pain with a NRS score ≥ 7/10 within the first 24 h after the termination of the axillary plexus block.

### 2.3. Statistical Analysis

#### 2.3.1. Power Analysis

The sample size was calculated based on the incidence of rebound pain. The presence of RP was defined as pain intensity score > 7 (NRS, 0 to 10) reported by the patient after axillary plexus block resolution [6]. Based on values of RP incidence after peripheral nerve block resolution approaching 45% to 50% [5,6] and assuming that the incidence of RP with intravenous DEXA administration would be reduced by half and thereby would approach 25% [18], a minimum of 46 patients was needed in each group to have an alpha value of 0.05 and a power of 0.8.

#### 2.3.2. Data Analysis

For analysis of retrospective data, the intravenous DEXA dose was separated into high (HD; >0.1 mg/kg) or low (LD; <0.1 mg/kg) doses. DEXA HD and LD patients were compared to control patients (*n* = 55) included in a previous randomized controlled trial [6] regarding demographics (age, gender parity and BMI) to ensure adequate matching.

Statistical analysis was performed with SigmaStat 3.5 (Systat Software GmbH, Erkrath, Germany). Results were expressed as proportions, mean ± standard deviation or median value (interquartile range) as specified. According to a Kolmogorov–Smirnov normality test, parametric data between the groups were compared by an unpaired Student t-test and nonparametric data by Mann–Whitney and Kruskal–Wallis Rank Sum tests. Categorical data were compared using the chi-squared test and Fisher exact test using a two-tailed probability. A *p*-value less than 0.05 was considered to be significant. A backward stepwise regression model (*p* < 0.05 significant) was also be used to test the predictive value of intravenous DEXA prevention on the development of RP.

## 3. Results

Between January 2021 and January 2022, 210 patients underwent elective upper limb surgery under axillary plexus block, and intraoperative IV DEXA administration was noticed in 133 of these patients. A total of 118 patients were included in the retrospective analysis (Figure 1, Flow diagram).

As reported in Table 1, these patients were comparable regarding age, sex and type of surgery to the control group where patients did not received intraoperative DEXA (patients included in a previous prospective randomized study on RP, *n* = 55) [6]. Intraoperative administration of IV DEXA was associated with a significant reduction of the pain felt when PNB wore off as well as to a significant reduction of RP incidence (23% versus 47%, *p* = 0.002) (Table 1). The total duration of PNB however was not influenced by IV DEXA administration at the doses used. The DEXA doses ranged from 0.05 to 0.12 mg/kg.

Thereafter, for statistical analysis, patients who received IV DEXA were divided into a high-dose DEXA group (DEXA HD, dose > 0.1 mg/kg, *n* = 64) and a low-dose DEXA group (DEXA LD, dose < 0.1 mg/kg, *n* = 54) as described in Table 2. By comparison with the control group (i.e., no DEXA administration), intraoperative DEXA reduced the occurrence of RP. We observed a dose-related trend which, however, was not significant. Similarly, a DEXA dose-related trend to less pain felt when PNB wore off was noticed by the patients, but it was not either statistically significant.

At the DEXA doses used, no significant impact on the duration of the axillary block was observed. Postoperative pain scores assessed at 24 h after surgery were not affected by the dose of intraoperative DEXA.

The characteristics of patients who had received IV DEXA and presented with and without RP phenomenon are presented in Table 3. The main differences were higher BMI, higher average preoperative pain score at the surgical site, higher catastrophizing score and higher incidence of patients defined as “high catastrophizers” (score > 23/52, i.e., >75th percentile). Bone surgery also was more frequent in RP patients. The dose of IV DEXA did not differ between patients with and without rebound pain.

When considering the full population of patients (*n* = 173, including the control group), a positive correlation was noted between the intensity of RP and the intensity of preoperative pain (0.445, *p* = 0.000) as well as for the level of preoperative catastrophizing score (0.283, *p* = 0.000). Important factors associated with the presence of RP (*p* value < 0.05) were entered in a Backward Stepwise Regression model including the intraoperative administration of DEXA independently of the dose used. In the final model, bone surgery (*p* < 0.001), high catastrophizing (*p* < 0.001) and the absence of intraoperative DEXA (*p* = 0.027) were predictive of RP occurrence when the axillary PNB wore off.

## 4. Discussion

The present results support the intraoperative use of intravenous DEXA (dose 0.05–0.12 mg/kg) for the prevention of rebound pain after upper limb surgery under axillary plexus block. Regardless the dose of IV DEXA administered, our study showed a significant decrease in RP occurrence (23% vs. 47%; *p* = 0.002). This finding is in agreement with previous reports [5,13]. However, at the doses we used, a dose-dependent effect of DEXA on the occurrence of RP could not be found.

Several studies have assessed the use of perineural DEXA to increase the duration of sensory nerve block, mostly the interscalene plexus block, and to improve postoperative analgesia after upper limb procedures. These studies have shown a dose-dependent effect with a ceiling effect for doses higher than 4 mg [19,20]. Because the rebound pain phenomenon has increased as a subject of interest, some studies have recently evaluated the preventive effect of perineural DEXA. After shoulder surgery, perineural DEXA 5 mg reduced RP occurrence from 83% to 37% [11], and perineural DEXA 8 mg decreased RP from 48.8% to 11% [12]. Despite the effectiveness of perineural DEXA, that route of administration is still considered off-label due to potential neurotoxicity [9].

Intravenous DEXA also potentiates PNB and increases the duration of the sensory block. Equipotent doses between perineural and IV routes have been questioned. From published meta-analyses, perineural DEXA seems more effective to prolong PNB analgesia, but no greater difference is observed between both routes when DEXA doses of 8 mg and higher are used [21]. Regarding lower doses of IV DEXA, Desmet and colleagues found a dose-dependent effect on PNB duration after shoulder surgery but only a significant effect for doses higher than 2.5 mg DEXA (i.e., 0.03 mg/kg) [22]. Studies assessing the preventive effect of IV DEXA on RP incidence are still scarce. First, a large retrospective cohort study [5] which included different types of blocks in both upper and lower limbs procedures reported a beneficial effect for an average DEXA dose of 6 mg (range: 4–20 mg). Second, a prospective randomized study including 51 adult patients scheduled for hand surgery found a reduction of RP within the first postoperative 36 h from 50% in the placebo group to 9% in the 16 mg (i.e., around 0.23 mg/kg) IV DEXA group [13]. To the best of our knowledge, the present study is the first to assess a dose-related preventive effect of low IV DEXA doses on RP after axillary PNB in ambulatory patients.

According to the aforementioned findings, a dose-related preventive effect of IV DEXA on RP might be questioned. However, if we compare the incidence of RP in our DEXA-LD (<0.1 mg/kg) with that of RP in Holmberg’s study (0.23 mg/kg), the difference is not statistically significant (14/54 [26%] vs. 2/23 [9%], *p* = 0.13, Fisher exact two-tailed). Similarly, if we compare RP incidence in our DEXA-HD group (20%) with that in Holmberg’s study (9%), there is no statistically significant difference (*p* = 0.33) [13]. A ceiling effect in DEXA RP preventive effect should also be taken into account. Based on the fact that perineural and intravenous DEXA doses > 8 mg are equipotent to prolong PNB duration and analgesic effect [21], no significant difference in RP incidence could be found regarding IV DEXA-HD in our patients (0.10–0.12 mg/kg: 13/64 = 20%) versus IV DEXA 16 mg (0.23 mg/kg: 2/23 = 9%) [13] versus perineural DEXA 8 mg (7/63 = 11%) [12]. These results further question the mechanisms underlying the DEXA preventive effect on RP.

Prolonged nerve block, hence a smoother recovery of nociceptive sensations, has been proposed as a mechanism to reduce RP [13]. At the doses used in our patients, we did not observe an increase in the duration of PNB what is in opposition with previous studies. In example, IV DEXA 16 mg significantly prolonged the axillary block analgesia [13], and Desmet reported a time extension to the first analgesic request after PNB at an IV DEXA dose as low as 0.03 mg/kg [22]. In contrast to the aforementioned studies, our study was retrospective and powered to assess the rebound pain incidence and not the PNB duration. It is worth noting that the sensory block duration may not affect the RP phenomenon as previously underlined [5].

Two studies in healthy volunteers [23,24] using a moderate dose of IV DEXA (4 mg) did not observe a prolongation of nerve block duration but pointed out the fact that benefits observed in patients probably rely on the anti-inflammatory effect of IV DEXA [25]. A previous meta-analysis about intravenous DEXA has suggested that only doses higher than 0.1 mg/kg demonstrate analgesic effects which are not dose-related [15]. Our results show a reduction of both pains felt when PNB wears off and RP incidence at IV DEXA doses is lower than 0.1 mg/kg, independent of the axillary block duration. The individual sensitivity to the anti-inflammatory effect of glucocorticoids is variable as observed for the response to other analgesics with anti-inflammatory properties. Among the risk factors of RP, bone surgery and high catastrophizing score are well known [5,6]. Both risk factors interact with inflammatory processes that may be involved in RP.

Bone surgery leads to the local release of pro-inflammatory cytokines, such as interleukin 6 (IL-6), which activate and sensitize sensory nerves, leading to an amplified pain signal [26]. More, these pro-inflammatory mechanisms may be exacerbated in some patients, enhancing postoperative pain and hyperalgesia [27]. For example, a higher level of catastrophizing is associated with greater reactivity of inflammatory mediators (e.g., IL-6) [28] which suggests that cognitive and emotional responses during the experience of pain may shape the pro-inflammatory responses of the immune system to noxious stimulation. The involvement of inflammatory mechanisms in RP may explain the predisposition to RP in patients with a high catastrophization score. Therefore, by reducing the inflammatory cascade, DEXA may help to reduce hyperalgesia when the PNB wears off.

There are some limitations of this study. First, its retrospective nature involving a single center. Additionally, data regarding DEXA administration and data regarding the control group were collected in different cohorts. Second, the follow-up of DEXA patients was limited to the first 24 h, which could have contributed to the loss of additional data. In the literature, late RP, i.e., at 36 and even 48 h, is reported. Finally, the doses of IV DEXA used were left to the discretion of the anesthesiologists in charge of the patients, which may have led to possible distribution bias due to common practices and habits.

It is worth noting that no adverse effects in relation to the administration of DEXA were found in the patients’ files. The relatively low doses of DEXA used in our study may actually have contributed to the absence of glycemic disorders in the patients [29]. Similarly, we did not record any cases of perineal irritation in the patients included in our study which is probably due to a slow administration of DEXA (a common practice within our team of anesthetists) [30].

In conclusion, our results support the administration of intraoperative IV DEXA as a preventive measure to reduce the occurrence of RP.

To our knowledge, this study is the first dedicated to investigating the dose-dependent effect of intraoperative DEXA (low doses used to prevent PONV and to improve postoperative analgesia) in the prevention of RP. We found a preventive effect on RP including at very low doses (<0.1 mg/kg) and independent of the PNB duration. A comparison with the existing literature may be in favor of an IV DEXA ceiling effect on RP prevention which contrasts with the IV DEXA dose-related effect on sensory block duration. Further prospective studies should confirm the present findings and investigate the mechanisms underlying the IV DEXA preventive effect on rebound pain.

## Figures and Tables

**Table 1:** Comparison between control group and DEXA ^1^ group.

	Control Group (*n* = 55)	DEXA Group (*n* = 118)	*p* Value
Age (years)	52 ± 18	45 ± 16	0.428
Sex ratio (female/male) (*n*)	29/26	78/40	0.298
BMI ^2^ (kg/m^2^)	25 (21–29)	26 (22–29)	0.266
Bone surgery (*n*)	21 (39%)	64 (40%)	0.854
Tourniquet duration (min)	22 (14–38)	21 (12–33)	0.742
Preoperative			
Catastrophizing (0–52)	12 (3–23)	11 (3–22)	0.774
Central sensitization (0–36)	9.0 (6–12)	8.5 (4–15)	0.449
Preoperative pain			
Average pain (NRS 0–10)	2.0 (0–4)	2.0 (0–5)	0.403
Maximal pain (NRS 0–10)	4.5 (1–8)	5.0 (0–7)	0.510
Night pain (NRS 0–10)	0 (0–3)	0 (0–4.5)	0.764
DN4 ^3^ value (0–10)	3.0 (1–4)	2.0 (1–4)	0.622
PNB ^4^ duration			
Total duration (min)	630 (506–795)	640 (410–905)	0.892
Pain when PNB wears off (NRS ^5^ 0–10)	4.5 (2–8)	3.3 (1–6) *	0.030
Incidence of rebound pain (NRS >7/10) (*n*)	26 (47%)	27 (23%) *	0.002

^1^ Dexamethasone; ^2^ Body Mass Index; ^3^ Douleur Neuropathique en 4 questions; ^4^ Peripheral Nerve Block; ^5^ Numerical Rating Scale; * *p* Value ≤ 0.05.

**Table 2:** Effect of intraoperative DEXA ^1^ administration (LD ^2^ & HD ^3^) on PNB ^4^ outcomes.

	Control Group (*n* = 55)	DEXA LD (*n* = 54)	DEXA HD (*n* = 64)	*p* Value
DEXA dose (mg/kg)	----	0.06 (0.05–0.07)	0.10 (0.10–0.12)	<0.001
PNB duration				
H1–H2 ^5^ (min)	400 (309–541)	292 (195–540)	332 (223–577)	0.092
H2–H3 ^6^ (min)	180 (120–307)	240 (165–300)	205 (79–405)	0.331
Total duration (min)	630 (506–795)	583 (445–825)	661 (402–960)	0.650
Preoperative pain at day 1				
Average pain (NRS ^7^ 0–10)	25 (1–55)	2.0 (1–4)	2.0 (0–5)	0.408
Maximal pain (NRS 0–10)	4.0 (2–7)	6.0 (2–8)	5.0 (1–8)	0.564
Pain when PNB wears off (NRS 0–10)	4.5 (2–8)	4.0 (1–7)	3.0 (1–6)	0.053
Incidence of rebound pain (NRS > 7/10) (*n*)	26 (47%)	14 (26%) *	13 (20%) *	0.029

* *p* < 0.05 with control group: ^1^ Dexamethasone; ^2^ Low Dose; ^3^ High Dose; ^4^ Peripheral Nerve Block; ^5^ Time interval between the time of the end of the block (H1, day and time) and the beginning of the onset of the paresthesia reported by the subject (H2, day and time after the block); ^6^ Time interval between the time of beginning of the occurrence of paresthesia reported by the subject (H2, day and time after block) and finally the onset of pain at surgery site (H3, day and time after block); ^7^ Numerical rating scale.

**Table 3:** Characteristics of patients with and without rebound pain among patients who received intraoperative DEXA 1 (*n* = 118).

	Rebound Pain (*n* = 27)	No Rebound Pain (*n* = 91)	*p* Value
Age (years)	45 ± 16	51 ± 18	0.073
Sex female (*n*)	20 (74%)	48 (53%)	0.075
BMI ^2^ (kg/m^2^)	28 ± 6 *	25 ± 5.5	0.026
Bone surgery (*n*)	17 (65%) *	31 (34%)	0.013
Tourniquet duration (min)	34 ± 26	24 ± 17	0.121
Preoperative			
Catastrophizing (0–52)	20 (4–37) *	9 (2–21)	0.017
High catastrophizers (*n*)	11 (42%) *	14 (16%)	0.007
Central sensitization (0–36)	8.5 (2–17)	8.5 (4–15)	0.787
Central sensitization positive (*n*)	4 (16%)	12 (14%)	0.757
Preoperative pain			
Average pain (NRS ^3^ 0–10)	4.5 (2–6) *	2.0 (0–4)	0.009
Maximal pain (NRS 0–10)	7.3 (1–9)	4.5 (0–7)	0.065
Night pain (NRS 0–10)	1 (0–6)	0 (0–3.8)	0.222
DN4 ^4^ value (0–10)	3.0 (2–4.5)	2.0 (1–4)	0.135
PNB ^5^ duration			
Total duration (min)	630 (506–795)	640 (410–905)	0.892
Pain when PNB wears off (NRS ^3^ 0–10)	8.0 (7–8.9) *	2.0 (1–4)	<0.001
DEXA dose (mg/kg)	0.08 (0.06–0.10)	0.09 (0.06–0.10)	0.650

* *p* < 0.05 with control group ^1^ Dexamethasone; ^2^ Body Mass Index; ^3^ Numerical rating score; ^4^ Douleur Neuropathique en 4 questions; ^5^ Peripheral Nerve Block.

## Data Availability

The data presented in this study are available on request from the corresponding author and the Supplementary Materials.

## Supplementary Materials

The following supporting information can be downloaded at: https://www.mdpi.com/article/10.3390/jcm12134310/s1, File S1: STROBE Statement—Checklist of items that should be included in reports of cohort studies.
